# Acute oxalate nephropathy induced by oral high-dose vitamin C alternative treatment

**DOI:** 10.1093/ckj/sfu013

**Published:** 2014-03-02

**Authors:** Louis-Denis Poulin, Julie Riopel, Vincent Castonguay, Fabrice Mac-Way

**Affiliations:** 1 Division of Nephrology, CHU de Québec, L'Hôtel-Dieu de Québec Hospital and Faculty of Medicine, Laval University, Quebec, QC, Canada; 2 Division of Pathology, CHU de Québec, L'Hôtel-Dieu de Québec Hospital and Faculty of Medicine, Laval University, Quebec, QC, Canada; 3 Division of Haematology-Oncology, CHU de Québec, L'Hôtel-Dieu de Québec Hospital and Faculty of Medicine, Laval University, Quebec, QC, Canada

**Fig. 1.**
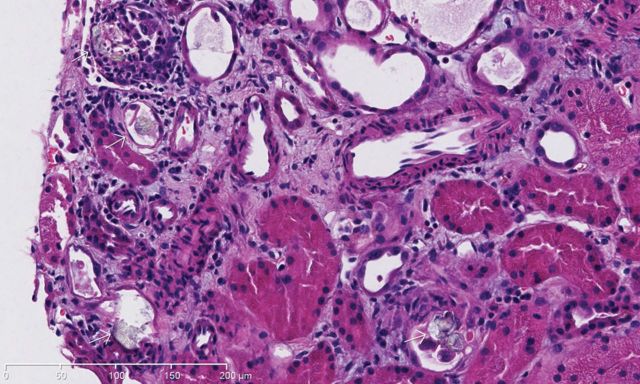
Intra-tubular deposition of calcium oxalate crystals (white arrows) and acute tubular injury in adjacent tubules. H&E ×20.

**Fig. 2.**
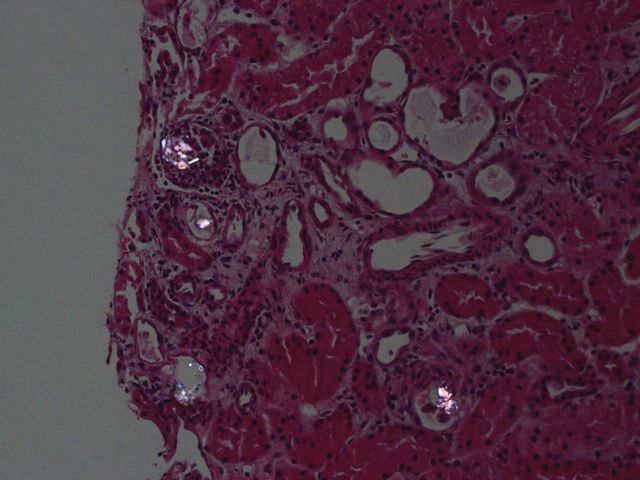
Birefringent calcium oxalate crystals under polarized light. H&E ×20.

A 59 year-old woman presented for abdominal pain, fatigue and decreased urine output. Eight months before she underwent a hysterectomy with bilateral salpingo-oophorectomy for a uterine leiomyosarcoma but was diagnosed 6 months later with local recurrence. She refused radiotherapy and palliative chemotherapy but rather preferred alternative treatment with oral high-dose vitamin C. Acute renal failure (ARF) was diagnosed (creatinine 166 versus 48 µmol/L 2 months before) and urinalysis revealed abundant calcium oxalate. Abdominal scan did not reveal renal obstruction. Renal biopsy showed extensive tubular calcium oxalate crystal deposition with some degree of acute tubular injury and mild interstitial inflammation with eosinophils (Figures 1 and 2). She denied any ethylene glycol ingestion and she was not known for bypass surgery or malabsorption. Hemodialysis treatments were initiated to increase creatinine and for hypervolemia but unfortunately, her condition slowly deteriorated. After 3 weeks of hemodialysis, the patient's renal function has not recovered and she was finally transferred to palliative care unit. Oxalate nephropathy is mostly seen with ethylene glycol intoxication, malabsorption or in primary hyperoxaluria. Vitamin C is metabolized into oxalate and can induce ARF by precipitation of calcium oxalate in renal tubules when excessive dose is taken. The patient reported that she was taking over 4 g of vitamin C daily during 30 consecutive days. Our case combined to recently published papers [1–3] underline the importance for clinicians to increase their awareness about this potential but easily preventable complication in patients taking high-dose vitamin C as alternative treatment.
